# A Cross-Sectional Survey Study on the Diagnosis and Management of Critical Illness-Induced Corticosteroid Insufficiency in Saudi Arabia

**DOI:** 10.7759/cureus.49982

**Published:** 2023-12-05

**Authors:** Rayan Abubakker Qutob, Abdullah Alaryni, Abdullah Alghamdi, Eysa N Alsolamy, Khalid Al Harbi, Yousef Alammari, Abdulrahman Mohammed Alanazi, Abdalmohsen A Ababtain, Osamah Ahmad Hakami, Ziyad Abdullah Aleid, Abdullah Saud Alhaqbani, Rahaf Abdulkhaliq Alshehri, Bedoor Abdulaziz Almoshiqeh, Almonther Qusai Alhejazi

**Affiliations:** 1 Department of Internal Medicine, College of Medicine, Imam Mohammad Ibn Saud Islamic University, Riyadh, SAU; 2 Department of Critical Care Medicine, King Abdullah bin Abdulaziz University Hospital at Princess Nourah bint Abdulrahman University, Riyadh, SAU; 3 Department of Internal Medicine, King Abdullah Medical City in Holy Capital (KAMC-HC), Makkah, SAU; 4 College of Medicine, Imam Mohammad Ibn Saud Islamic University, Riyadh, SAU

**Keywords:** survey, saudi arabia, insufficiency, diagnosis, corticosteroid

## Abstract

Background

The presence of critical illness-induced corticosteroid insufficiency (CIRCI) is correlated with elevated concentrations of circulating biomarkers associated with inflammation and coagulation in multiple domains. The management of adrenal insufficiency remains a topic of ongoing debate and disagreement among endocrinologists and intensivists. This study aimed to assess the extent of understanding regarding CIRCI among endocrinologists and intensivists who are actively practicing in Saudi Arabia.

Methods

This is an online cross-sectional survey study that was conducted between June and August 2023 to assess knowledge of CIRCI among endocrinologists and intensivists working in Saudi Arabia. The questionnaire tool for this study was constructed based on a previous literature review. Binary logistic regression analysis was used to define factors that affect participants’ knowledge of CIRCI.

Results

A total of 76 physicians were involved in this study. Around 32.9% (n= 25) of the participants described CIRCI correctly as an impairment of the hypothalamic-pituitary axis during critical illness. Around 35.5% (n=27) of the participants identified that widespread use of corticosteroids in critically ill patients prompted the need to revisit the concept, diagnosis, and management of CIRCI, and a similar proportion of the participants (35.5%) (n=27) identified that the role of corticosteroids in the management of CIRCI in critically ill patients may be beneficial in selected cases. Around 42.1% (n=32) of the participants identified that CIRCI is specific to critically ill patients while AI can occur in any individual. Around 17.1% (n=13) of the participants confirmed that there is no task force agreement on whether corticosteroids should be used in adult patients with sepsis but without shock. The mean knowledge score of the study participants was 3.6 (sd: 2.2) out of 10, which demonstrates a weak level of knowledge of CIRCI (36.0%). Binary logistic regression analysis identified that physicians from the southern and western regions were less likely to be knowledgeable of CIRCI compared to physicians from the central region (p< 0.05).

Conclusion

The study revealed that the level of familiarity with CIRCI among endocrinologists and intensivists in Saudi Arabia fell short of the desired benchmark. Clinicians may opt to utilize delta cortisol levels following cosyntropin administration and random plasma cortisol levels as diagnostic measures for CIRCI, instead of relying on plasma-free cortisol or salivary cortisol levels in conjunction with plasma total cortisol. Adherence to customized treatment protocols is crucial to attain the most favorable results for patients.

## Introduction

Critical illness-related corticosteroid insufficiency (CIRCI) refers to the development of central adrenal insufficiency in long-term critically ill patients [1]. The prevalence of this condition exhibits variation based on the extent and length of its manifestation. The incidence of adrenal insufficiency in critically ill patients is anticipated to range from 0% to 30%, while in patients with septic shock, it may vary from 25% to 40% [2]. CIRCI is associated with increased levels of circulating biomarkers related to inflammation and coagulation across various aspects, including disease severity, morbidity, length of stay in the intensive care unit, and mortality [3]. The condition known as CIRCI is a complex and commonly observed phenomenon, and our comprehension of it continues to evolve [4]. It is imperative for critical care professionals to possess a comprehensive understanding of the pathophysiology and treatment of CIRCI due to the mounting body of evidence indicating its occurrence in various critical conditions [3]. A contentious issue exists about the establishment of a diagnostic test that would effectively and reliably identify CIRCI. Nevertheless, the delta cortisol, which refers to the shift in cortisol levels from baseline, is less than 9 μg/dL after 60 minutes of administering cosyntropin at a dosage of 250 μg. Physicians may consider using the use of random plasma cortisol levels below 10 μg/dL. Previous studies have proposed a serum cortisol level of 15 g/dL as a diagnostic criterion for adrenal insufficiency (AI) in individuals with critical illness [3,5]. In relation to the therapy of severe sepsis and septic shock, new guidelines suggest that a cortisol level of 18 g/dL is a suitable threshold for initiating glucocorticoid treatment [5]. Previous study has indicated possible advantages associated with this therapeutic approach; however, further investigation is required to ascertain the impact of the presence or absence of CIRCI on patients' response to corticosteroid therapy in such situations [6]. Due to a lack of knowledge among numerous clinicians in the appropriate management of adrenal insufficiency, there is a consequential occurrence of significant delays in the administration of treatment. It is imperative that healthcare practitioners have proper education and training on the subject of adrenal insufficiency. Additionally, patients should be well-equipped to handle the initial phases of emergency management [7]. There is a scarcity of research conducted in this particular field. In a study conducted in India by Sathya (2006), an assessment was made of the knowledge, attitudes, and practices of endocrinologists and intensivists with regard to adrenal insufficiency in critically ill patients. The findings indicated a lack of consensus among both endocrinologists and intensivists regarding the management of adrenal insufficiency in this population [8]. Hence, the aim of this study was to evaluate the level of knowledge of CIRCI among endocrinologists and intensivists practicing in Saudi Arabia.

## Materials and methods

Study design and settings

This is an online cross-sectional survey study that was conducted between June and August 2023 to assess knowledge of CIRCI among endocrinologists and intensivists working in Saudi Arabia.

Study population and recruitment

Study participants were recruited using the convenient sampling technique. This particular sampling technique is classified as non-probability sampling. The current investigation involved participants who met predetermined criteria for inclusion and indicated their willingness to participate in the research. At the initiation of the survey, participants were provided with an informed consent form, giving them the choice to either continue their participation in the study or decide to discontinue it. The research aims were comprehensively communicated to patients to improve their comprehension of the importance of their participation. The invitation letter for the study presented a comprehensive outline of the criteria for admission. The questionnaire tool was distributed to endocrinologists and intensivists working in Saudi Arabia through the Google Forms website to be filled out electronically. The inclusion criteria were Saudi and non-Saudi endocrinologists and intensivists who are currently working in Saudi Arabia. The exclusion criteria are non-physicians, physicians not working in Saudi Arabia, non-endocrinologists, non-intensivists, and participants who do not agree to participate in the survey.

Study tool

The questionnaire tool for this study was constructed based on a previous literature review [1,3]. The questionnaire tool collected data on participants’ demographic and practice characteristics such as age, gender, nationality, specialty, level of experience, years of experience, region of practice, and place of practice (see Appendices). The second section examined participants’ knowledge of CIRCI and comprised 10 multiple-choice questions. The knowledge section examined the participants in terms of the definition of CIRCI, conditions that prompted the need to revisit the concept, diagnosis, and management of CIRCI, the role of corticosteroids in the management of CIRCI in critically ill patients, how CIRCI differs from AI, diagnostic tests used for CIRCI, whether corticosteroids should be used in adult patients with sepsis but without shock, which corticosteroid regimen is recommended in patients with septic shock refractory to fluids and moderate vasopressor therapy, the minimum recommended duration of corticosteroid therapy required for septic shock, whether corticosteroids are recommended for patients with major trauma, and when should testing for recovery of the hypothalamic-pituitary-adrenal (HPA) axis be done after a prolonged ICU stay (≥ 4 weeks). For each correct answer, the participants were given a score of one, with a maximum attainable score of 10. The higher the score, the more knowledgeable the participant is.

Piloting of the questionnaire tool

The questionnaire tool underwent evaluation and validation by two medical specialists. Participants were asked to provide feedback on the clarity, comprehensibility, and face validity of the questions, as well as any challenges they found in understanding them. In addition, the participants were instructed to submit comments regarding any inquiries that they found to be disagreeable. Moreover, a preliminary study was undertaken utilizing a small number of the intended participants to evaluate their understanding of the survey tool before its widespread distribution.

Ethical approval

Ethical approval for this study was received from the Institutional Review Board at Al-Imam Mohammad Ibn Saud Islamic University, Riyadh, Saudi Arabia (reference number: 497/2023). Due to the retrospective nature of the research, and the use of data anonymously, the requirement for informed consent is requested to be waived by the ethical committee.

Data management and statistical analysis

Data were analyzed using the Statistical Package for Social Science software, version 29 (IBM Corp., Armonk, NY, USA) Frequency and percentages were used to display categorical variables. Mean and standard deviation (sd) were used to present continuous variables as the knowledge score was normally distributed. The mean knowledge score of the study participants (which is 3.6 (sd: 2.2)) was used to define the dummy variable for the binary logistic regression analysis, which was used to define factors that affect participants’ knowledge of CIRCI. The odds ratio was presented with a 95% confidence interval. The level of significance was set at 0.05.

## Results

Characteristics of the study participants

A total of 76 physicians were involved in this study. More than half of them were males (69.7%; n= 53). Around 44.7% (n= 34) of the participants were aged 25-34 years. The majority of the participants were Saudis (80.3%; 61) and were intensivists (75.0%; n= 57). Almost one-third of the participants were consultants (31.6%; 24). Around 47.4% (n= 36) of the participants had an experience of less than five years. Almost one-third of the study participants were practicing their profession in the central region (35.5%; n= 27) and working at a Ministry of Health secondary hospital (32.9%; n= 25). For further details on the characteristics of the study participants, refer to Table 1.

**Table 1:** Characteristics of the study participants Categorical data are presented as frequencies and percentages

Variable	Frequency	Percentage
Gender		
Males	53	69.7%
Females	23	30.3%
Age categories		
25-34 years	34	44.7%
35-44 years	23	30.3%
45-54 years	11	14.5%
55-64 years	4	5.3%
65 years and older	4	5.3%
Nationality		
Saudi	61	80.3%
Specialty		
Intensivist	57	75.0%
Endocrinologist	19	25.0%
Level of experience		
Registrar	14	18.4%
Senior registrar	8	10.5%
Resident	20	26.3%
Fellow	10	13.2%
Consultant	24	31.6%
Years of experience		
Less than 5 years	36	47.4%
5-10 years	20	26.3%
11-15 years	7	9.2%
16-20 years	7	9.2%
21 years and above	6	7.9%
Region of practice		
Central region	27	35.5%
Northern region	1	1.3%
Southern region	13	17.1%
Eastern region	21	27.6%
Western region	14	18.4%
Place of practice		
Private hospital	11	14.5%
University hospital	13	17.1%
Medical City or specialized hospital	15	19.7%
Ministry of Health secondary hospital	25	32.9%
Military of National Guard hospital	12	15.8%

Participants' knowledge of CIRCI

Table 2 presents participants’ responses to knowledge items that measured their knowledge of CIRCI. Around 32.9% (n= 25) of the participants described CIRCI correctly as an impairment of the hypothalamic-pituitary axis during critical illness. Around 35.5% (n= 27) of the participants identified that the widespread use of corticosteroids in critically ill patients prompted the need to revisit the concept, diagnosis, and management of CIRCI. Around 40.8% (n=31) of the participants confirmed that delta cortisol after cosyntropin is a diagnostic test used for CIRCI.

**Table 2:** Participants’ knowledge of CIRCI ICU: intensive care unit; AI: adrenal insufficiency; CBG: cortisol-binding globulins; CIRCI: critical illness-induced corticosteroid insufficiency; § right answer

Variable	Frequency	Percentage
Which of the following describes CIRCI?		
Primary adrenal insufficiency in the ICU setting	34	44.7%
Impairment of the hypothalamic-pituitary axis during critical illness	25	32.9%
Excessive cortisol clearance during critical illness	9	11.8%
Elevated cortisol binding globulins (CBG) in critical illness §	8	10.5%
Which of the following conditions prompted the need to revisit the concept, diagnosis, and management of CIRCI?		
Widespread use of corticosteroids in critically ill patients §	27	35.5%
Emergence of new diagnostic tests for CIRCI	26	34.2%
Increased understanding of adrenal gland physiology	12	15.8%
Advances in cortisol measurement techniques	11	14.5%
What is the role of corticosteroids in the management of CIRCI in critically ill patients?		
They may be beneficial in selected cases of CIRCI §	27	35.5%
They are the primary treatment for CIRCI	24	31.6%
They have no impact on CIRCI	14	18.4%
They are used to confirm the diagnosis of CIRCI	11	14.5%
How does CIRCI differ from adrenal insufficiency (AI)?		
CIRCI is specific to critically ill patients, while AI can occur in any individual §	32	42.1%
CIRCI has an underlying adrenal pathology, while AI can have other causes	19	25.0%
CIRCI and AI are synonymous terms	13	17.1%
CIRCI is a permanent condition, while AI is transient	12	15.8%
Although there’s no consensus, which of the following is a diagnostic test used for CIRCI?		
Delta cortisol after cosyntropin §	31	40.8%
Plasma free cortisol	17	22.4%
Salivary cortisol	15	19.7%
Urinary free cortisol	13	17.1%
Should corticosteroids be used in adult patients with sepsis but without shock?		
No, they are not beneficial	27	35.5%
It depends on the severity of sepsis	20	26.3%
Yes, they are beneficial	16	21.1%
There is no task force agreement §	13	17.1%
Which corticosteroid regimen is recommended in patients with septic shock refractory to fluids and moderate vasopressor therapy?		
Hydrocortisone < 400 mg/day §	35	46.1%
Prednisolone < 40 mg/day	16	21.1%
Dexamethasone < 24 mg/day	15	19.7%
Methylprednisolone < 60 mg/day	10	13.2%
What is the minimum recommended duration of corticosteroid therapy required for septic shock?		
≥ 3 days §	27	35.5%
≥ 7 days	21	27.6%
≥ 5 days	18	23.7%
≥ 14 days	10	13.2%
Are corticosteroids recommended for patients with major trauma?		
No, they are not beneficial §	27	35.5%
It depends on the severity of the trauma	21	27.6%
Yes, they are beneficial	16	21.1%
There is no task force agreement	12	15.8%
After a prolonged ICU stay (≥ 4 weeks), when should testing for recovery of the hypothalamic-pituitary-adrenal (HPA) axis be done?		
6 weeks after ICU discharge	28	36.8%
1 week after ICU discharge §	20	26.3%
12 weeks after ICU discharge	20	26.3%
6 months after ICU discharge	8	10.5%

Around 17.1% (n= 13) of the participants confirmed that there is no task force agreement on whether corticosteroids should be used in adult patients with sepsis but without shock. Around 46.1% (n=35) of the participants reported that hydrocortisone < 400 mg/day is recommended in patients with septic shock refractory to fluids and moderate vasopressor therapy. Around 35.5% (n=27) of the participants reported that the minimum recommended duration of corticosteroid therapy required for septic shock is more than or equal to three days. Around 26.3% (n=20) of the participants reported that one week after ICU discharge from prolonged ICU stay (≥ 4 weeks) testing for recovery of the hypothalamic-pituitary-adrenal (HPA) axis should be done.

Predictors of physicians’ knowledge

The mean knowledge score of the study participants was 3.6 (sd: 2.2) out of 10; which demonstrates a weak level of knowledge of CIRCI (36.0%). Binary logistic regression analysis identified that physicians from the southern and western regions were less likely to be knowledgeable of CIRCI compared to physicians from the central region (p< 0.05) (Table 3).

**Table 3:** Predictors of physicians’ knowledge *p<0.05

Variable	Odds ratio of being knowledgeable (95% confidence interval)	P-value
Gender		
Females (Reference group)	1.00	
Males	1.81 (0.66-4.97)	0.253
Age categories		
25-34 years (Reference group)	1.00	
35-44 years	2.68 (0.90-8.02)	0.078
45-54 years	1.19 (0.30-4.68)	0.803
55-64 years	-	
65 years and older	-	
Nationality		
Non-Saudi (Reference group)	1.00	
Saudi	0.91 (0.29-2.82)	0.867
Specialty		
Intensivist (Reference group)	1.00	
Endocrinologist	1.15 (0.41-3.26)	0.790
Level of experience		
Registrar (Reference group)	1.00	
Senior registrar	0.45 (0.08-2.67)	0.379
Resident	0.61 (0.16-2.43)	0.487
Fellow	0.32 (0.06-1.79)	0.195
Consultant	0.64 (0.17-2.40)	0.502
Years of experience		
Less than 5 years (Reference group)	1.00	
5-10 years	2.10 (0.69-6.39)	0.191
11-15 years	1.87 (0.36-9.59)	0.455
16-20 years	0.56 (0.10-3.28)	0.521
21 years and above	0.28 (0.03-2.65)	0.267
Region of practice		
Central region (Reference group)	1.00	
Northern region	-	
Southern region	0.15 (0.03-0.79)	0.025*
Eastern region	1.30 (0.41-4.16)	0.658
Western region	0.22 (0.05-0.96)	0.045*
Place of practice		
Private hospital (Reference group)	1.00	
University hospital	0.78 (0.14-4.27)	0.772
Medical city or specialized hospital	0.64 (0.12-3.41)	0.598
Ministry of Health secondary hospital	3.72 (0.84-16.47)	0.084
Military or National Guard hospital	1.25 (0.23-6.72)	0.795

The Appendices present the items used to examine the participants' CIRCI knowledge.

## Discussion

Critical illness-related corticosteroid insufficiency refers to the development of central adrenal insufficiency in patients who are critically ill for a prolonged period [1]. This condition is associated with elevated levels of inflammatory and coagulation-related biomarkers, which have been found to affect disease severity, morbidity, duration of intensive care unit stay, and mortality. As a result, it is crucial for critical care professionals to possess a comprehensive understanding of the pathophysiology and treatment of CIRCI, as a growing body of evidence indicates its occurrence in various critical conditions [3]. Therefore, the objective of this study was to assess the knowledge level of CIRCI among endocrinologists and intensivists practicing in Saudi Arabia.

The study findings indicate that participants had a low level of knowledge regarding CIRCI, with an average knowledge score of 3.6 out of 10. Binary logistic regression analysis revealed that physicians in the southern and western regions were statistically less likely to possess knowledge of CIRCI compared to their counterparts in the central region. This lack of knowledge among physicians may be attributed to the absence of precise diagnostic criteria for identifying patients who do not exhibit an elevation in cortisol levels during acute illness [9]. It is important to note that the level of total cortisol does not accurately reflect steroid levels, as it is influenced by free cortisol and its interaction with cortisol receptors. These receptors undergo alterations during acute stress as an adaptive mechanism for survival [10]. Furthermore, a research study examining the clinical practices and patient outcomes related to CIRCI in patients with refractory shock revealed the existence of a noteworthy threshold that must be exceeded before considering CIRCI as a potential cause of shock. The study also suggested that the apparent underdiagnosis of CIRCI could be attributed to a reduced frequency of steroid administration in cases where there was a justifiable suspicion of CIRCI in patients experiencing shock [11].

Approximately 32.9% of the study participants demonstrated a precise understanding of CIRCI as a dysfunction of the hypothalamic-pituitary axis during critical illness. It is observed that critical illness stimulates the activation of the hypothalamic-pituitary-adrenal (HPA) axis, resulting in the secretion of cortisol. However, prolonged activation of the HPA axis can lead to elevated cortisol levels (hypercortisolemia) as well as decreased cortisol levels (hypocortisolemia), both of which can negatively impact the recovery process [12]. The HPA axis is known to have a significant impact on the stress response, as it regulates various physiological processes such as metabolism, cardiovascular function, and the immune system [13]. However, it has been observed that HPA axis dysfunction is a common occurrence in patients with systemic inflammation [14]. In such cases, alterations in HPA axis activity can lead to changes in the regulation of cortisol, metabolism, and carrier protein levels. Consequently, the interpretation of cortisol levels in critically ill patients differs from that in healthy individuals. These differences present limitations that may result in the misinterpretation of adrenal sufficiency status in critically ill patients [5].

Furthermore, a significant proportion of the participants (approximately 35.5%) recognized the necessity to reevaluate the concept, diagnosis, and management of CIRCI due to the widespread utilization of corticosteroids in critically ill patients. Similarly, an equivalent percentage of participants (35.5%) acknowledged the potential benefits of corticosteroid administration in managing CIRCI in specific cases. It was also found that the adverse effects associated with corticosteroid usage are influenced by variables such as dosage, administration method, and treatment duration, particularly within the intensive care unit (ICU) setting, particularly during short-term interventions for CIRCI [14]. However, recent research suggests that stress doses of corticosteroids have a dual effect. They suppress systemic inflammation by decreasing the transcription of proinflammatory mediators while also preserving innate and Th1 immune responsiveness. This dual action is beneficial in preventing an overwhelming compensatory anti-inflammatory response [15,16]. However, the impact of corticosteroids on interleukin (IL)-10 and soluble tumor necrosis factor (TNF) receptors is still uncertain. Therefore, it is recommended to avoid using corticosteroids in chronically critically ill patients in the ICU who are believed to have a compensatory anti-inflammatory response [14,17-19].

Furthermore, a significant proportion of the participants (approximately 42.1%) acknowledged that CIRCI is specific to critically ill patients. On the other hand, AI can occur in any individual. It is worth noting that AI is considered a serious condition that can arise from various pathological conditions in individuals [20]. It is important to highlight that the inability to effectively manage acute AI-related illness or infection can lead to a life-threatening adrenal crisis [21]. This suggests that the severity of the illness plays a crucial role in determining its criticality. Additionally, approximately 40.8% of the study participants confirmed that the measurement of delta cortisol after cosyntropin administration is a diagnostic test used for CIRCI. However, according to the Guidelines for the Diagnosis and Management of CIRCI in critically ill patients published by the Society of Critical Care Medicine (SCCM) and the European Society of Intensive Care Medicine in 2017, there is no consensus on a reliable diagnostic test for CIRCI. Nevertheless, clinicians may opt to utilize delta cortisol following cosyntropin administration and random plasma cortisol levels as diagnostic measures for CIRCI, instead of relying on plasma-free cortisol or salivary cortisol levels over plasma total cortisol. This choice is made because the total cortisol level does not adequately reflect steroid levels, as it is influenced by free cortisol and its interaction with cortisol receptors [22]. During episodes of acute stress, it has been observed that these receptors undergo alterations as a means of adapting to the situation to enhance survival [10].

The study findings revealed that approximately 17.1% of the participants acknowledged the lack of consensus among task forces regarding the use of corticosteroids in adult patients with sepsis but without shock. This perception of the absence of task force agreement stems from conflicting evidence. On one hand, certain studies suggest that corticosteroids may have a beneficial effect on severe sepsis and septic shock, as indicated by systematic reviews demonstrating improved survival rates [22]. On the other hand, other studies conclude that glucocorticoids should not be administered to septic patients, as two well-conducted trials failed to demonstrate any benefits and even suggested increased mortality in certain subgroups [23]. Indeed, the contentious issue surrounding the use of corticosteroid therapy for septic shock has been recognized [24]. Consequently, it is recommended that short durations of high-dose corticosteroids should be avoided while extended administration of stress doses of hydrocortisone may potentially decrease the requirement for vasopressors and enhance the reversal of shock [25]. Furthermore, a study revealed that approximately 46.1% of the participants indicated that the recommended dosage of hydrocortisone for patients with septic shock that is unresponsive to fluids and moderate vasopressor therapy is less than 400 mg per day. Additionally, the study found evidence suggesting that the utilization of low-dose hydrocortisone could potentially have positive effects in the treatment of septic shock, including the reduction of recovery time [26]. Furthermore, approximately 35.5% of the participants indicated that the minimum duration of corticosteroid therapy recommended for septic shock is three days or longer. This contrasts with the findings of our study, which suggest that extended therapy with lower doses of corticosteroids (5-7 days or more) may have potential benefits in the treatment of septic shock [27].

In our study, a significant proportion (approximately 35.5%) of the participants expressed the view that the administration of corticosteroids, which are commonly recommended for patients with major trauma, does not yield any benefits. This aligns with the prevailing notion that corticosteroids should not be administered as a routine treatment for patients with severe head injuries, as there is a lack of evidence supporting their efficacy in the context of acute traumatic injury [28,29]. Additionally, it was observed among the study participants that approximately 26.3% of them advocated for the implementation of the HPA axis recovery testing one week after discharge from the ICU following a prolonged ICU stay of four weeks or more. This recommendation stems from the understanding that prolonged stimulation of the HPA axis can result in either hypercortisolemia or hypocortisolemia, both of which can hinder the recovery process in individuals suffering from critical illnesses [12]. Hence, there is a pressing necessity to conduct tests for the evaluation of HPA axis recovery. The short synacthen test (SST) is a viable method for assessing the restoration of HPA axis function [30]. Furthermore, it is imperative to closely monitor and evaluate the recuperation of the HPA axis in patients with CIRCI [31]. This is due to the potential ramifications of HPA axis dysfunction on their capacity to effectively respond to stressors, such as infections or other medical challenges [32].

Several recommendations can be derived from the study findings about CIRCI among endocrinologists and intensivists in Saudi Arabia. First and foremost, there exists a pressing demand for improved education regarding CIRCI, with a particular emphasis on the southern and western regions where knowledge levels have been comparatively lower. Additionally, it is imperative to develop clear diagnostic criteria that may include the incorporation of delta cortisol following cosyntropin administration, to effectively address the existing limitations. Furthermore, with respect to the utilization of corticosteroids, healthcare professionals had to meticulously evaluate the appropriate dosage, administration approach, and length of therapy while carefully assessing the possible advantages in the management of illnesses such as CIRCI and septic shock. Besides, it is imperative to closely monitor the emerging body of research about the utilization of corticosteroids in particular medical circumstances such as sepsis in the absence of shock. In addition, it is crucial to adhere to personalized treatment strategies for optimal patient outcomes. Furthermore, it is imperative to acknowledge the particularity of CIRCI in relation to patients who are critically sick, as this understanding is crucial for carrying out further evaluations, particularly following extended periods of time spent in the intensive care unit. The assessment of the HPA axis's recovery, maybe using the SST, is of utmost importance in the evaluation of patients' capacity to respond to stresses after being discharged. These recommendations aim to improve overall CIRCI management and awareness among healthcare professionals in Saudi Arabia.

This study has limitations. The cross-sectional study design prevented us from examining causality across the variables of the study. The online survey tool might have restricted the generalizability of our study findings due to the possibility of sampling bias.

## Conclusions

The level of knowledge on CIRCI among endocrinologists and intensivists in Saudi Arabia was found to be below the optimal standard. Clinicians may choose to employ delta cortisol levels subsequent to cosyntropin treatment and random plasma cortisol levels as diagnostic tools for CIRCI, rather than depending on plasma-free cortisol or salivary cortisol levels in relation to plasma total cortisol. Patients who are critically ill may suffer from relative adrenal insufficiency, and administering external corticosteroids could assist in regulating the inflammatory response and enhancing outcomes.

## Appendices

**Table 4:** Questionnaire items

Variable	Answer
Which of the following describes CIRCI?	
Impairment of the hypothalamic-pituitary axis during critical illness	
Elevated cortisol binding globulins (CBG) in critical illness	
Primary adrenal insufficiency in the ICU setting	
Excessive cortisol clearance during critical illness	
Which of the following conditions prompted the need to revisit the concept, diagnosis, and management of CIRCI?	
Widespread use of corticosteroids in critically ill patients	
Emergence of new diagnostic tests for CIRCI	
Increased understanding of adrenal gland physiology	
Advances in cortisol measurement techniques	
What is the role of corticosteroids in the management of CIRCI in critically ill patients?	
They are the primary treatment for CIRCI	
They may be beneficial in select cases of CIRCI	
They are used to confirm the diagnosis of CIRCI	
They have no impact on CIRCI	
How does CIRCI differ from adrenal insufficiency (AI)?	
CIRCI is specific to critically ill patients, while AI can occur in any individual	
CIRCI is a permanent condition, while AI is transient	
CIRCI and AI are synonymous terms	
CIRCI has an underlying adrenal pathology, while AI can have other causes	
Although there’s no consensus, which of the following is a diagnostic test used for CIRCI?	
Delta cortisol after cosyntropin	
Plasma free cortisol	
Urinary free cortisol	
Salivary cortisol	
Should corticosteroids be used in adult patients with sepsis but without shock?	
There is no task force agreement	
No, they are not beneficial	
It depends on the severity of sepsis	
Yes, they are beneficial	
Which corticosteroid regimen is recommended in patients with septic shock refractory to fluids and moderate vasopressor therapy?	
Hydrocortisone < 400 mg/day	
Dexamethasone < 24 mg/day	
Prednisolone < 40 mg/day	
Methylprednisolone < 60 mg/day	
What is the minimum recommended duration of corticosteroid therapy required for septic shock?	
≥ 3 days	
≥ 5 days	
≥ 7 days	
≥ 14 days	
Are corticosteroids recommended for patients with major trauma?	
It depends on the severity of the trauma	
No, they are not beneficial	
Yes, they are beneficial	
There is no task force agreement	
After a prolonged ICU stay (≥ 4 weeks), when should testing for recovery of the hypothalamic-pituitary-adrenal (HPA) axis be done?	
1 week after ICU discharge	
6 weeks after ICU discharge	
12 weeks after ICU discharge	
6 months after ICU discharge	
